# Urinary Metabolomic Profile of Preterm Infants Receiving Human Milk with Either Bovine or Donkey Milk-Based Fortifiers

**DOI:** 10.3390/nu12082247

**Published:** 2020-07-27

**Authors:** Marzia Giribaldi, Chiara Peila, Alessandra Coscia, Laura Cavallarin, Sara Antoniazzi, Sara Corbu, Giulia Maiocco, Stefano Sottemano, Francesco Cresi, Guido E. Moro, Enrico Bertino, Vassilios Fanos, Flaminia Cesare Marincola

**Affiliations:** 1 CREA Research Centre for Engineering and Agro-Food Processing, 10135 Turin, Italy; marzia.giribaldi@crea.gov.it; 2 Neonatal Unit, University of Turin, City of Health and Science of Turin, 10126 Turin, Italy; chiara.peila@unito.it (C.P.); giulia.maiocco@edu.unito.it (G.M.); stefano.sottemano@unito.it (S.S.); francesco.cresi@unito.it (F.C.); enrico.bertino@unito.it (E.B.); 3 Institute of the Science of Food Production—National Research Council, 10095 Grugliasco (TO), Italy; laura.cavallarin@ispa.cnr.it (L.C.); sara.antoniazzi@ispa.cnr.it (S.A.); 4 Department of Chemical and Geological Sciences, Cittadella Universitaria, University of Cagliari, 09042 Cagliari, Italy; sari.crb@gmail.com (S.C.); flaminia@unica.it (F.C.M.); 5 Italian Association of Human Milk Banks (AIBLUD), 20126 Milan, Italy; guidoemoro@tiscali.it; 6 Neonatal Intensive Care Unit, Neonatal Pathology and Neonatal Section, Azienda University Polyclinic, University of Cagliari, 09124 Cagliari, Italy; vafanos@tin.it

**Keywords:** adjustable fortification, bovine milk, donkey milk, ^1^H NMR, human milk, preterm, protein fortifiers, urinary metabolome

## Abstract

Fortification of human milk (HM) for preterm and very low-birth weight (VLBW) infants is a standard practice in most neonatal intensive care units. The optimal fortification strategy and the most suitable protein source for achieving better tolerance and growth rates for fortified infants are still being investigated. In a previous clinical trial, preterm and VLBW infants receiving supplementation of HM with experimental donkey milk-based fortifiers (D-HMF) showed decreased signs of feeding intolerance, including feeding interruptions, bilious gastric residuals and vomiting, with respect to infants receiving bovine milk-based fortifiers (B-HMF). In the present ancillary study, the urinary metabolome of infants fed B-HMF (*n* = 27) and D-HMF (*n* = 27) for 21 days was analyzed by ^1^H NMR spectroscopy at the beginning (T0) and at the end (T1) of the observation period. Results showed that most temporal changes in the metabolic responses were common in the two groups, providing indications of postnatal adaptation. The significantly higher excretion of galactose in D-HMF and of carnitine, choline, lysine and leucine in B-HMF at T1 were likely due to different formulations. In conclusion, isocaloric and isoproteic HM fortification may result in different metabolic patterns, as a consequence of the different quality of the nutrients provided by the fortifiers.

## 1. Introduction

The achievement of optimal growth is one of the main targets for the successful management of preterm infant care [1]. Inadequate nutrition and/or poor postnatal growth have been reported as negatively associated with neurocognitive outcomes in preterm infants [2]. Furthermore, an inadequate nutritional management of preterm newborns may increase the risk of developing cardiovascular and metabolic diseases in adult life, such as dyslipidemia, insulin resistance and type 2 diabetes [3]. Thus, provision of optimal nutrition in the neonatal period, particularly for very-low birth weight (VLBW, <1500 g) infants, has become a priority not only for achieving optimal short-term outcomes, but also for preventing long-term complications.

Although human milk (HM) is undoubtedly the gold standard of nutrition for every newborn, in the case of premature birth, it is inadequate for the nutritional needs of infants since it provides insufficient amounts of some nutrients. HM should therefore be supplemented (fortified) with specific nutrients, and particularly with protein, calcium and phosphate [1,4]. Although HM fortification is widely used in neonatal intensive care units all over the world, preterm infants receiving fortification often still experience suboptimal growth and feeding intolerance. Thus, during the last decade, new fortification strategies [5] and different fortifiers [6] have been evaluated, in the effort of minimizing adverse effects and improving growth rates. Further, the effects of different levels of fortification on short-term growth and their impact on metabolic responses of preterm infants have been topics of discussion. Nevertheless, the optimal method for HM fortification still remains to be determined, and a variety of protocols are currently used.

In short-term nutritional studies [7,8,9,10], metabolomics has shown to be a promising investigating tool. Metabolomics is a discipline aimed at characterizing the metabolome, i.e., the pools of low-molecular weight metabolites (<1.5 kDa) present in cells, tissues, organs or biological fluids [11]. This class of compounds may be of endogenous and exogenous origin. The formers are the end-products of the gene expression, while the latter arise from external sources such as diet, drug or environmental exposure. Thus, metabolomics studies can lead to define the biochemical phenotype of a cell or tissue and the impact of factors such as genotype, environment, lifestyle and diet. In nutritional studies, metabolomics contributions have so far allowed the identification of metabolic signatures of diets and enhanced the understanding of how dietary components may influence metabolic pathways [12]. The biological fluids used for this purpose are urine and blood. The former is particularly suitable for studying nutrient intake or identifying food-specific biomarkers, while the latter is a reliable indicator of physiological response to food. Most of the metabolomics applications in neonatal nutrition research have been focused on deepening the knowledge about the HM composition, while a smaller number of investigations have assessed the impact of nutrition on infant metabolism [13,14,15,16,17]. Among these, to our knowledge, there is only one study on preterm infants that has explored the urinary metabolomics profile of infants fed with diets supplemented by two different levels of extra nutrients [13].

Recently, we have shown that preterm and VLBW infants receiving isocaloric and isoproteic supplementation of HM with either bovine milk-based fortifier (B-HMF) or experimental donkey milk-based fortifiers (D-HMF) achieve similar auxological outcomes. Additionally, a donkey milk-based fortifier significantly reduced the occurrence of feeding intolerance, feeding interruptions, bilious gastric residuals and vomiting [18]. As an extension of our previous study, the current investigation aims at applying the metabolomics approach to explore the modulation of the two above-mentioned fortifiers on the metabolic phenotype of preterm infants after a 21-day period of adjustable (ADJ) fortification. Due to its characteristics and simple non-invasive methods of collection, urine is particularly suited for metabolomics studies on preterm infants, offering the possibility to monitor the global system of the whole organism without hazard effects for newborns. Since donkey milk has a protein profile more similar to that of human milk in terms of relative abundance and primary structure [19] in comparison with bovine milk, we hypothesized that such differences may impact the protein utilization in preterm infants and, consequently, may result in a different metabolic response.

## 2. Materials and Methods

### 2.1. Clinical Trial and Intervention

The study was conducted in accordance with the Declaration of Helsinki and performed in the NICU of the University, City of Health and Science of Turin. The study was approved by the local ethics committee (AN: 0025847, 27 May 2014), and registered (http://www.isrctn.com/ISRCTN70022881, ISRCTN70022881). Informed written consent was obtained from parents before enrolment. The recruitment period was 27 November 2014 to 22 December 2016. All the details about the clinical trial have been deeply described in two previous reports [18,20]. The present research was performed in a subpopulation of the original cohort that successfully completed the study period and whose urinary samples at the beginning of fortification (Time 0) and 21 days later (Time 1) were available.

### 2.2. Study Population

Fifty-four premature infants with gestational age < 32 weeks and/or birth weight ≤ 1500 g were enrolled in the present study. Exclusion criteria are detailed in [20]. After written informed parental consent was obtained, the population of newborns was randomly divided into two groups at the start of fortification (enteral feeding volume ≥ 80 mL/kg/day reached within the first 4 weeks of life):

▪ Bovine—Human Milk Fortifier (B-HMF) Group (*n* = 27) receiving ADJ fortification with commercial multi-component fortifier (FM85 Nestlè) and protein concentrate (Protifar Nutricia), derived from bovine milk, for a minimum of 21 days;
▪ Donkey—Human Milk Fortifier (D-HMF) Group (*n* = 27) receiving ADJ fortification with multi-component fortifier and protein concentrate derived from donkey milk, not commercially available, and prepared according to current EU legislation on foods for special medical purposes, for a minimum of 21 days.

The composition of component fortifiers and protein concentrates are reported in Supplementary Table S1.

### 2.3. Urine Sample Collection

Urine samples were collected from each enrolled infant at two time points: the day when fortification was started (T0) and 21 days after the beginning of fortification (T1). Specimens were collected non-invasively using a sterile cotton ball placed in the disposable diaper. In the absence of fecal contamination, about 1.5 mL of urine was aspired with a syringe and transferred to a sterile 2 mL vial. Samples were then frozen immediately, stored at −80 °C and shipped on dry ice to the University of Cagliari.

### 2.4. Sample Preparation

Urine samples were thawed in ice. To avoid any possible bacterial growth during the preparation, an aliquot of 8 μL of a 1% aqueous solution of NaN_3_ was added to 800 μL of urine. The samples were then centrifuged at 12,000× *g* for 10 min at 4 °C to remove any solid particle, and 630 μL of the supernatant solution were mixed with 70 μL of 1.5 M phosphate buffer solution (pH 7.4) containing trimethylsilylpropanoic acid (TSP, final concentration 1 mM). The mixture was vortexed, and 650 μL were transferred into a 5 mm wide NMR tube.

### 2.5. H NMR Measurements and Data Processing

^1^H NMR spectra were recorded at 300 K using a Varian Unity Inova 500 MHz NMR spectrometer (Agilent Technologies, Santa Clara, CA, USA) operating at 499,839 MHz. Water resonance was suppressed by pre-saturation during the first increment of the NOESY pulse sequence with irradiation occurring during the 2 s relaxation delay and the 1 ms mixing time. NMR spectra were acquired with a 90° pulse, an acquisition time of 1.5 s and 256 scans collected in 64k data points over a spectral width of 6000 Hz.

^1^H NMR spectra were processed using the MestReNova program (version 14.0.1, Mestrelab Research SL, Santiago de Compostela, Spain). After Fourier transformation with 0.3 Hz line broadening, ^1^H spectra were phased and baseline-corrected, and the chemical shift scale was set by assigning a value of δ = 0.00 ppm to the signal for the internal standard TSP. Identification of NMR peaks was done according to the literature [21,22], Human Metabolome database (http://www.hmdb.ca) and the Chenomx NMR suite 8.1 software (evaluation version, Chenomx, Edmonton, Canada). Figure S1 reports peak attributions.

Spectra were aligned to compensate for the shift of the signals of some metabolites due to small inter-sample pH changes. Then, they were uniformly binned to 0.0025 ppm intervals between 0.5 and 9.5 ppm, excluding the region corresponding to water (4.6–5.2 ppm) and TSP (−0.5–0.5 ppm) signals. Bins were normalized to the total spectral area to compensate the different dilutions of original urine samples.

### 2.6. Statistical Analyses

Multivariate statistical data analysis was performed by using SIMCA version 16.1 (Umetrics, Umea, Sweden). Prior to analysis, the data matrix was pretreated using Pareto scaling. The data were then analyzed using the following multivariate statistical analysis (MVA) techniques: principal component analysis (PCA) and orthogonal partial least squares discriminant analysis (OPLS-DA).

PCA, an unsupervised exploratory analysis, was performed to explore preliminarily the structure of the data matrix and the presence of outliers. PCA models were evaluated through the correlation coefficient R^2^ and the prediction coefficient Q^2^. R^2^ is defined as the percentage of variance in the data set explained by the model and indicates the goodness of the fitting. Q^2^ is defined as the percentage of variance of the data set predicted by the model and indicates the goodness of the prediction. Both R^2^ and Q^2^ may have a value between 0 and 1. The R^2^ and Q^2^ values were calculated based on 7-fold cross-validation. Satisfactory values of R^2^ and Q^2^ must be ≥0.5, with |R^2^ − Q^2^| < 0.2–0.3. 

The OPLS-DA technique is the orthogonal implementation of the partial least squares-discriminant analysis (PLS-DA) regression, used to maximize the correlation between two sets of variables (X and Y) by reducing the data into a few latent variables. It is applied where a dummy variable Y matrix is used. This technique improves the interpretation of the spectroscopic variations between the discriminated groups by removing information that has no effect on separation. The quality of the OPLS-DA models was evaluated based on the fitness (R^2^Y) and prediction (Q^2^Y) abilities determined through the default leave 1/7th out cross-validation. Additionally, the robustness of the models was assessed by calculation of cross-validation ANOVA (CV-ANOVA) and y-table permutation testing over 400 iterations [23]. Potential variables that were statistically significant for the group discrimination were identified by analyzing the S-line correlation coefficient plot. Variables were selected according to a *p*(corr) ≥ 0.5 and *p*(cov) ≥ 0.05. The correlation coefficient *p*(corr) refers to the credibility of the contribution of the variable in the mathematical model, while the coefficient *p*(cov) denotes the modelled covariation.

The univariate statistical analysis was performed by the GraphPad Prism Statistics software package, version 8.1.2 (GraphPad Prism Software Inc., San Diego, CA, USA). Student’s *t*-test was used for the comparison of between-group data. The prevalence of specific characteristics/morbidities/outcomes was compared between the two subpopulations of mothers and infants by means of relative risk (RR) analysis. A probability level of *p* < 0.05 was considered statistically significant for univariate statistics.

## 3. Results

The characteristics of preterm infants, of their mothers and of the prevalence of common physiopathological events for the population under investigation are reported in Table 1. Table 2 reports the main clinical outcomes following ADJ fortification in the two groups. The relative risk (RR) ratio for the primary outcome of the original clinical study [18], i.e., the occurrence of at least one feeding intolerance episode, was calculated in the subpopulation. The RR ratio for feeding intolerance, defined as interruption of enteral feeding for at least eight consecutive hours during the observation period, was found to be 0.40 in the D-HMF group, equal to that reported in the main clinical trial. Significant differences for the two groups were found for the time needed to reach a full enteral feeding and for the occurrence of breastmilk at discharge, with the D-HMF group reaching the goal two days before B-HMF, and thus having a higher prevalence (1.58 RR) of infants receiving breastmilk.

Urinary metabolomics patterns were then investigated. PCA was performed on the NMR dataset to explore any natural groupings of samples and to identify possible outliers. Since all urine spectra collected at T0 were dominated by the intense peaks of gluconate (administered as Ca-gluconate in the parenteral nutrition solution before fortification) (Figure S1), the NMR signals of this compound were removed from the data set prior to MVA to enhance the contribution of less abundant metabolites to the spectral profile. 

Initially, PCA was applied only to samples collected at time T0 to verify the homogeneity of the two preterm groups at the beginning of ADJ fortification. The absence of discernible patterns and subgroups in the scores plot of the first two principal components (PCs) confirmed that the two classes were homogeneous (Figure S2). Based on Hotelling’s T2 test at 95% confidence and DModX test, three samples were identified as strong outliers, and two as moderate outliers. The spectra of all five samples were characterized by the presence of intense unassigned peaks at 5.5 and 7.5 ppm. Nevertheless, since no spectral anomaly was observed, all outliers were kept for the subsequent MVA.

**Figure 1:**
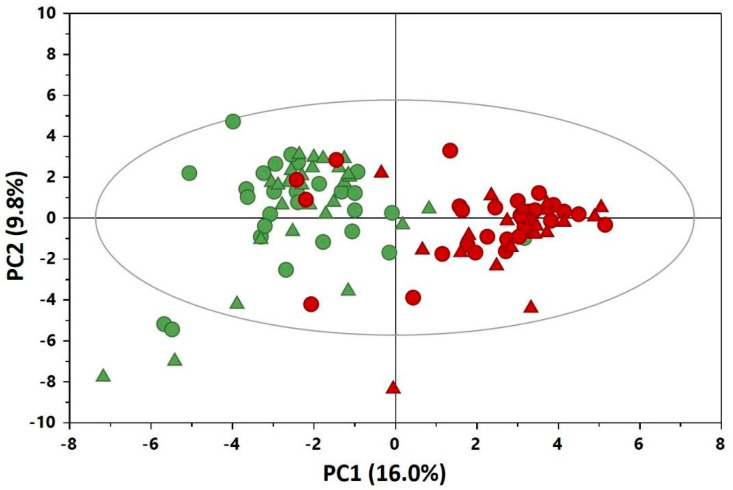
Principal component (PC) 1 vs. PC2 scores plot of the principal component analysis (PCA) model derived from the ^1^H NMR spectra of urine collected from preterm infants before (green, T0) and at 21 days (red, T1) of adjustable (ADJ) fortification (R^2^X = 0.259; Q^2^ = 0.162): ●, B-HMF (infants receiving commercial multicomponent fortifier and protein concentrate derived from bovine milk); ▲, D-HMF (infants receiving experimental multicomponent fortifier and protein concentrates derived from donkey milk).

Figure 1 shows the PCA scores plot built with the whole NMR dataset. The first two PCs explained about 26% of the variation in the metabolic profile. No clustering of scores was shown in terms of the type of fortifier. On the other hand, despite the low values of R^2^ and Q^2^ (0.26 and 0.16, respectively), a distribution of samples was rather evident according to the sampling time, with specimens collected at time T0 mainly distributed on the left side of the plot and those collected at time T1 on the right side. 

**Figure 2:**
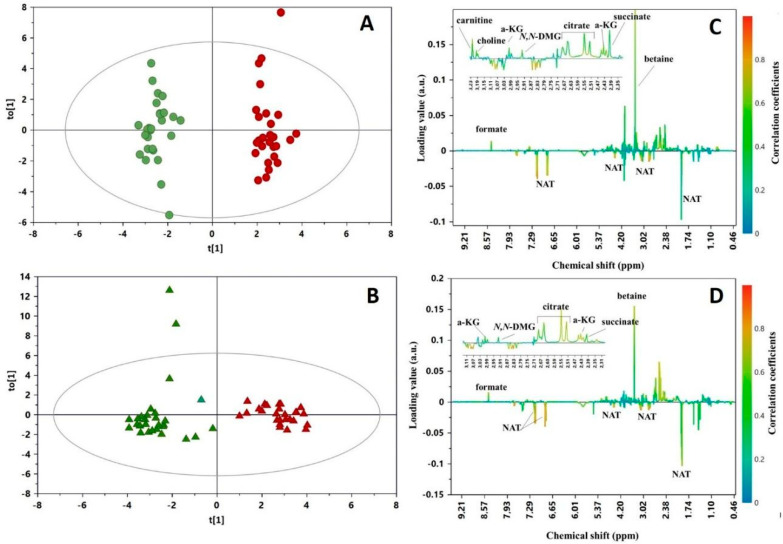
Scores (**left**) and S-line correlation coefficient (**right**) plots of the orthogonal partial least squares discriminant analysis (OPLS-DA) models built with urine of B-HMF (●, **A**,**B**) and D-HMF (▲, **C**,**D**) groups collected before (green, T0) and at 21 days (red, T1) from preterm infants receiving ADJ fortification. Statistical parameters of the models: (**A**,**B**) R^2^Y = 0.969; Q^2^ =0.734; *p* < 0.005; (**C**,**D**) R^2^Y = 0.889; Q^2^ = 0.786; *p* < 0.0001. The abbreviations used are as follow: α-KG, alpha-ketoglutarate; NAT, *N*-acetylthyrosine; *N*,*N*–DMG, *N*,*N*-dimethylglycine.

To examine the temporal changes of the urinary metabolome in relation to the type of fortification, an OPLS-DA model was built for each group of newborns in a pair-wise comparison of samples collected at the two time points (i.e., T0 vs. T1). Both models exhibited a clear discrimination between groups, as illustrated in Figure 2A,B. For the model built with urinary samples from the B-HMF group, we recorded R^2^Y = 0.896 and Q^2^ = 0.673, while for the model from the D-HMF group, we obtained R^2^Y = 0.871 and Q^2^ = 0.764. The validity and predictability of the models was confirmed by a *p*-value < 0.001, calculated by CV-ANOVA and a permutation test (the Q^2^ intercept value obtained from the regression line was −0.56 and −0.30 for B-HMF and D-HFM, respectively). By the analysis of the S-line correlation coefficient plot, the most influential metabolites contributing to the OPLS discrimination were identified (Figure 2C,D): carnitine, choline, betaine, *N*,*N*-dimethylglycine (*N*,*N*–DMG), alpha-ketoglutarate (α-KG), formate, citrate, succinate and *N*-acetylthyrosine (NAT). 

**Figure 3:**
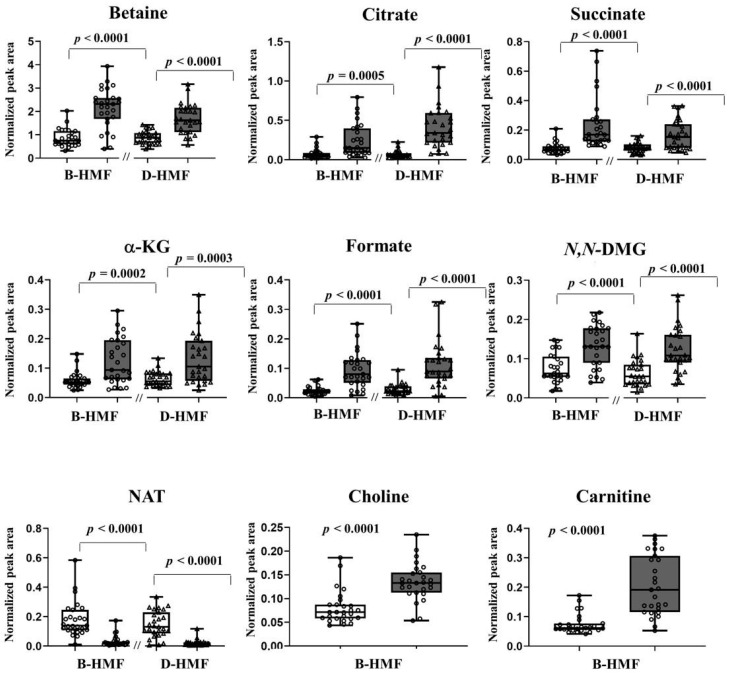
Box-plots indicating relative urine concentrations of discriminant metabolites (fold change > 2; *p* < 0.001) observed between the samples collected at T0 (white) and T1 (grey): ●, B-HMF: ▲, D-HMF.

In agreement with the models, univariate statistical analysis of the normalized intensities of the identified discriminant metabolites revealed several unique features exhibiting a high fold change (> 2) over the course of the intervention period, in combination with a high significance level (Figure 3). In particular, some similarities between the two fortifications were pointed out. They included significantly temporal increasing urinary levels of betaine (2.8 and 2.1 fold change for B-HMF and D-HMF, respectively, both *p* < 0.0001), citrate (fold change = 6.3 and *p* = 0.0005 for B-HMF; fold change = 8.1 and *p* < 0.0001 for D-HMF), succinate (3.1 and 2.6 fold change for B-HMF and D-HMF, respectively, both *p* < 0.0001), α-KG (fold change = 2.5 and *p* = 0.0002 for B-HMF; fold change = 2.5 and *p* = 0.0003 for D-HMF), formate (5.9 and 4.9 fold change for B-HMF and D-DHMF, respectively, both *p* < 0.0001) and *N*,*N*-DMG (2.4 and 2.5 fold change for B-HMF and D-HMF, respectively, both *p* < 0.0001). NAT decreased 0.27- and 0.60-fold in B-HMF and D-HMF, respectively (both *p* < 0.0001). Additionally, differently from D-HMF, the urinary metabolome of B-HMF was characterized by an increasing content of carnitine (fold change = 3.3, *p* < 0.0001), and choline (fold change = 3.3, *p* < 0.0001). 

**Figure 4:**
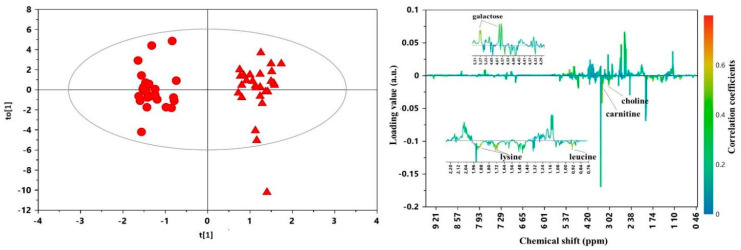
Scores (**left**) and coefficient loadings line (**right**) plots of the OPLS-DA model built with samples collected after 21 days (T1) of ADJ fortification: ●, B-HMF; ▲, DHMF. Statistical parameters of the models: R^2^Y = 0.950; Q^2^ = 0.576; *p* <0.005.

OPLS-DA was also applied for the pair-wise comparison between the urine of infants receiving the two types of ADJ fortification for 21 days (T1). The corresponding scores and coefficient loadings line plots are shown in Figure 4. For this model, we recorded R^2^Y = 0.950 and Q^2^ = 0.576, showing relevant metabolic differences between the two groups. In particular, the fortification of HM with a commercial bovine milk-based product resulted in significantly higher levels of carnitine, choline, lysine and leucine in B-HMF, as compared with the D-HMF group, while urine samples of D-HMF were significantly richer in galactose.

## 4. Discussion

Recently, we conducted a clinical trial [18] demonstrating that a better tolerance of D-HMF with respect to B-HMF was observed in a population of preterm and VLBW infants. We speculated that the quality of donkey milk proteins could be responsible for this better tolerance, the two diets being isoproteic and isocaloric, although differing in origin (bovine vs. donkey milk) and form (extensively hydrolyzed bovine whey proteins vs. whole donkey milk proteins). In addition, major differences in the two diets included the type of carbohydrate (maltodextrins vs. lactose), and the type and quantity of lipids. The aim of the present study was to extend our previous findings by examining the urinary metabolome of preterm infants receiving an exclusive HM diet (maternal and/or donated) fortified with extra nutrients, deriving either from bovine or donkey milk, according to an ADJ fortification protocol [1]. To the best of our knowledge, only Moltu and coworkers [13] have so far investigated the urinary metabolomics profile of preterm infants, whose diet was supplemented with extra nutrients. In their study, a standard fortification protocol was chosen and the diets of control vs. intervention groups were not balanced for caloric and protein intake. Standard and ADJ fortification intervention are not equal in the achievement of growth outcomes for preterm infants [1], with the first protocol more often resulting in undernutrition and sub-optimal growth [24]. 

The two populations monitored in the present study were similar to those reported in the main clinical trial. During intervention, the auxological outcomes were similar in the two groups, as previously observed. Feeding intolerance was lower in D-HMF, although not significantly different from those of B-HMF [18]. Further, as regards to the subpopulation included in the present metabolomics study, the total number of hours of feeding interruptions was lower in the D-HMF group, as well as the bile stagnation episodes, in accordance with the observations made on the whole trial population [18]. The D-HMF group reached full enteral feeds significantly earlier than B-HMF, with a higher number of infants receiving breastmilk at any amount at discharge, a parameter that was not included in the first report on the whole population [18]. 

In the study period under investigation (21 days of ADJ fortification), the two groups of recruited infants shared a common temporal urinary pattern. In particular, the metabolite profiling revealed for both fortifications an increase in betaine, citrate, formate, α-KG, *N,N*-DMG and succinate, and a decrease in NAT at T1 as compared with T0. In accordance with the literature [13,16,25], these changes are indicative of the normal postnatal metabolic adaptation pattern of preterm infants. 

NAT is a water-soluble tyrosine derivative, commonly used as a tyrosine source in parenteral nutrition. The high levels of NAT in the urine sampled before the beginning of ADJ fortification can be reasonably linked to the intravenous feeding started immediately after birth. Thus, the decrease in NAT contents observed after 21 days of fortification (T1) may be considered as a urinary marker of the expected progressive decrease in parenteral nutrition in preterm infants fed fortified HM, as specified in the fortification protocol.

Betaine and *N*,*N*-DMG are choline derivatives. Betaine is an important osmolyte which is also involved in one-carbon metabolism as a major source of methyl groups in mammals. It could have both a dietary and a metabolic origin. Betaine is also the major metabolite of choline excreted in urine, and its elevate excretion is often taken as an indication of renal failure. *N*,*N*-DMG is a one-carbon metabolite product of betaine metabolism. The temporal urinary increase in betaine observed in both groups of preterm infants is in good agreement with the results of previous studies [16,25,26]. Accordingly, this trend has been reported as a common response for preterm infants in the first weeks after birth, mainly due to kidney immaturity [26], and particularly relevant in neonates born at less than 34 weeks of gestation. 

Citrate, succinate, α-KG and formate are involved in the citric acid cycle. The citric acid cycle is a key metabolic pathway occurring in the matrix of the mitochondrion, generating most of the energy produced in cellular respiration. Changes in the citric acid cycle during the postnatal period had already been observed in previous studies [13,16,25]. In particular, in term infants, citrate excretion was found to be positively associated with height and weight [21], while in preterm newborns, it was linked to high energy requirements during rapid growth [13,16]. A high activation of citrate synthase as a consequence of a human or donkey milk-containing diet was also observed in young rats [27]. Taken together, the concomitant increased excretion of citric acid, cycle intermediates and compounds involved in choline metabolism, and the decrease in NAT after 21 days of intervention common to both groups, may represent a metabolic pattern of physiological maturation for the subjects fed fortified HM. 

Beside common responses, some discriminant signatures for bovine- and donkey-derived fortifiers were observed. A significant increase in urinary carnitine and choline was observed in B-HMF infants at T1, but not in D-HMF. Two amino acids, i.e., lysine and leucine, showed a significantly higher content in B-HMF than in D-HMF, while D-HMF was characterized by a higher urinary excretion of galactose. 

Carnitine plays an important role in fatty acid (FA) oxidation. It is responsible for the long-chain FA transport across the mitochondrial membrane, where they undergo β-oxidation to produce energy. Infants have decreased capacity of endogenous synthesis of carnitine and thus they are at risk of developing carnitine deficiency. Although term HM is a good source of carnitine [28], premature HM may not contain adequate carnitine concentrations. Thus, most infant formulas are supplemented to provide a carnitine content similar to those of term human milk [29,30]. Nevertheless, questions have been posed about the clinical advantage of adding carnitine to both short-term regimens of parenteral nutrition and to enterally fed infants [31,32]. Thus, in the present study, the higher urinary excretion of carnitine in the B-HMF-fed group may support the hypothesis that, when an exclusively HM diet is used, the target requirements of carnitine are satisfied by the HM supply itself [32], and that exceeding carnitine is excreted in urines, rather than being used as a carrier for FA oxidation. 

Choline is obtained from the diet or by sequential methylation of phosphatidylethanolamine. It is a precursor of membrane and lipoprotein phospholipids and the neurotransmitter acetylcholine, and plays a vital role in the human neonate for the developing brain. Human milk is known to contain higher amounts of choline than bovine milk, so that it is supplemented in the formula during manufacture. Choline content has been shown by ^1^H NMR to be significantly more abundant in preterm than in term HM [33] and in formula-fed newborns compared with breastfed infants [14]. As in the case of creatinine, the increased excretion of choline in the B-HMF group compared with the D-HMF group might indicate that the amount of choline that is needed for preterm and VLBW infants to meet the requirements is provided by HM itself. This is also indicated by the physiological increase in choline-related metabolites, such as betaine and *N*,*N*-DMG, in both fortified groups of infants. The exceeding intake of choline and creatinine were, therefore, excreted in the urine samples of infants fed with such added ingredients, that were, on the contrary, not supplemented by D-HMF.

Data on the plasma of preterm infants fed with protein hydrolysate formulas, standard formula and fortified breast milk have shown that their enzymatic immaturity affects the amino acid (AA) concentrations, the net protein utilization and the total AA and energy intake, and that the quality of the proteins can also impact the plasma AA concentrations [34,35,36,37]. Furthermore, because of the immature renal tubular reabsorption of AAs, an excessive protein intake could be responsible for an increased excretion of these compounds in urine [38]. Even though we did not characterize the AA composition of the two fortifiers, considering the similarity of the two groups under investigation in terms of immaturity characteristics (i.e., gestational and postnatal age) and protein and energy intakes, as well as the differences in form and type of proteins between the two fortifications [39], we do not rule out a possible role of AA compositions and of protein quality on the higher urinary levels of leucine and lysine observed in B-HMF as compared with D-HMF. Furthermore, very recently, a clinical trial [40] on term infants breastfed or fed with two different formulas demonstrated that, before the introduction of complementary foods, circulating AAs, including leucine and lysine, were more abundant in the serum metabolome of formula-fed infants, whose metabolic phenotype was also characterized by high levels of insulin and urea. This metabolic phenotype was not reverted by decreasing the protein content of the formulas, thus indicating that slow AA clearance is partially responsible for a low utilization of the proteins in the formula-fed infants, eventually resulting in the elimination of excess nitrogen through an insulin-mediated AA catabolism. In the light of these findings, another possible contribution to the increased excretion of leucine and lysine only in the B-HMF group may arise from a different metabolic phenotype with respect to D-HMF and, in particular, a lower utilization of nitrogen, similar to that described for formula-fed infants [40]. 

Similar conclusions relative to the different carbohydrate supply of the two fortifications can be made for the higher level of galactose in D-HMF infants as compared with B-HMF. Indeed, it is worth noting that the lactose content in the multi-component fortifier derived from donkey milk is ten times higher than that of the multi-component fortifier obtained from bovine milk (Table S1 in Supporting Information). Lactose is an important source of galactose, a key source of energy and a crucial structural element in complex molecules, which is particularly important for early human development. Since intact lactose was not found as differentially detected in the urine from the two groups, it is likely that lactose provided with D-HMF was used to fuel the infants’ metabolism. This latest observation may also provide interesting perspectives for future studies, since galactose, as well as lactose, are widely recognized as prebiotics. In such a perspective, microbiome analysis, coupled to fecal metabolome analysis, may provide further information about the differential shaping of intestinal microbiota communities in infants fed an exclusive HM diet supplemented with different protein fortifiers.

Our study bears two main limitations: (i) the relative sample size; and (ii) the lack of information on the AA composition of the experimental product based on donkey milk. Additionally, a more complete picture of the infant metabolome would have been achieved by analyzing also the blood profile. Nevertheless, this last point has been intentionally avoided due to the vulnerability of preterm infants in neonatal intensive care units, that requires the preferential use of non-invasive methods of analysis. The main strengths of this study are: (i) a good match between the neonatal characteristics of the two groups, including a similar prevalence of twins in each group, that were randomly assigned either separately to each arm of the study, or to the same arm; (ii) the overall organization of the trial, that provided substantial evidence of a higher tolerability of D-HMF with respect to B-HMF infants, as supported by the different investigated parameters [18,41]; (iii) the detailed knowledge of the experimental product based on donkey milk, including the processing and manufacturing, performed by the authors specifically for the clinical trial; and (iv) the use of a balanced fortification strategy, isocaloric and isoproteic, thus limiting the effect of nutrient imbalance on the present findings.

In conclusion, considering that multicomponent fortifiers used in the present study provide additional proteins and carbohydrates (besides minerals, vitamins and trace elements) for preterm HM supplementation, and the proportion of proteins and energy were similar, the differences observed between the urinary metabolome of the B-HMF and D-HMF groups can be attributed to the different nutrient composition of the two fortifiers. Replication in a larger cohort including more detailed information on protein quality and AA composition of the two fortifiers is an important next step.

## Figures and Tables

**Table 1:** Maternal and neonatal characteristics and clinical conditions at randomization.

	B-HMF ^1^ *n* = 27	D-HMF ^2^ *n* = 27	*p*-Value ^4^
Maternal characteristics ^3^			
Pregravidic BMI in kg/m^2^, mean (SD)	24.1 (5.3)	22.4 (3.7)	0.187 ^ST^
Weight gain in kg, mean (SD)	9.3 (4.1)	8.8 (4.9)	0.708 ^ST^
Age in years, median (IQR)	34 (30–40)	32 (29–38)	0.248 ^ST^
Chronic diabetes, *n* (%)	0	1 (3.7)	n.a.
Chronic hypertension, *n* (%)	2 (7.4)	2 (7.4)	1 ^RR^
Gestational diabetes, *n* (%)	6 (22.2)	6 (22.2)	1 ^RR^
Gestational hypertension, *n* (%)	7 (25.9)	5 (18.5)	0.511 ^RR^
Cesarean delivery, *n* (%)	22 (81.5)	21 (77.8)	0.735 ^RR^
Assisted reproductive technology, *n* (%)	8 (29.6)	3 (11.1)	0.083 ^RR^
**Neonatal characteristics ^3^**			
Male, *n* (%)	13 (48.1)	15 (55.6)	0.585 ^RR^
Twins, *n* (%)	11 (40.7)	9 (33.3)	0.572 ^RR^
Gestational age < 32 wk., *n* (%)	20 (74.1)	16 (59.3)	0.242 ^RR^
VLBW (<1500 g), *n* (%)	25 (92.6)	21 (77.8)	0.117 ^RR^
SGA, *n* (%)	8 (29.6)	10 (37)	0.563 ^RR^
Birth weight in g, mean (SD)	1174 (326)	1227 (302)	0.541 ^ST^
Birth weight in SDS, mean (SD)	−0.477 (1.092)	−0.720 (1.2)	0.442 ^ST^
Respiratory distress syndrome, n (%)	23 (85.2)	26 (96.3)	0.151 ^RR^
Recovered PDA, *n* (%)	8 (29.6)	4 (14.8)	0.533 ^RR^
Age at randomization in days, median (IQR)	10 (7–16)	9 (7–14)	0.799 ^ST^
Age at start intervention in days, median (IQR)	10 (7–15)	10 (7–18)	0.613 ^ST^

**^1^** B-HMF: infants receiving commercial multicomponent fortifier and protein concentrate derived from bovine milk. **^2^**D-HMF: infants receiving experimental multicomponent fortifier and protein concentrates derived from donkey milk. ^3^ IQR: interquartile range; SD: standard deviation; BMI: body mass index; VLBW: very low birth weight; SGA: small for gestational age; SDS: standard deviation score; PDA: patent ductus arteriosus. ^4^ n.a.: not assessed. ^RR^ relative risk analysis. ^ST^ Student *t*-test.

**Table 2:** Neonatal clinical outcomes and morbidities during the observation period.

Clinical Outcome and Morbidities ^3^	B-HMF ^1^ *n* = 27	D-HMF ^2^ *n* = 27	*p*-Value ^4^
Length of hospital stay in days, median (IQR)	48 (38–73)	44 (33–66)	0.532 ^ST^
Early sepsis, *n* (%)	6 (22.2)	2 (7.4)	0.117 ^RR^
Late sepsis, *n* (%)	3 (11.1)	2 (7.4)	0.638 ^RR^
Necrotizing enterocolitis, *n* (%)	0	0	n.a.
Weight at end intervention in g, mean (SD)	1505 (426)	1596 (324)	0.386 ^ST^
Weight gain during intervention in g, mean (SD)	424 (153)	450 (127)	0.518 ^ST^
Length at end intervention in cm, mean (SD)	39.8 (3.6)	41.2 (3.1)	0.163 ^ST^
Length gain during intervention in cm, mean (SD)	3.1 (1.3)	3.1 (1.0)	0.873 ^ST^
Feeding intolerance, *n* (%)	5 (18.5)	2 (7.4)	0.217 ^RR^
Feeding interruptions, total hours	300	183	n.a.
Vomiting, *n* (%)	13 (48.1)	11 (40.7)	0.583 ^RR^
Gastric residuals, *n* (%)	4 (14.8)	3 (11.1)	0.685 ^RR^
Bile stagnation episodes, total n	7	1	n.a.
Breast milk at discharge, *n* (%)	12 (44.4)	19 (70.4)	0.046 ^ST^
Parenteral nutrition at end intervention, *n* (%)	4 (14.8)	1 (3.7)	0.151 ^RR^
Days to full enteral feeding, median (IQR)	23 (17–31)	21 (13–24)	0.021 ^ST^

^1^ B-HMF: infants receiving commercial multicomponent fortifier and protein concentrate derived from bovine milk. ^2^ D-HMF: infants receiving experimental multicomponent fortifier and protein concentrates derived from donkey milk. ^3^ IQR: interquartile range; SD: standard deviation. ^4^ n.a.: not assessed. ^RR^ relative risk analysis. ^ST^ Student *t*-test. Significant differences at *p* < 0.05 are labelled in bold.

## Supplementary Materials

The following are available online at https://www.mdpi.com/2072-6643/12/8/2247/s1. Table S1: Composition of protein fortifiers used in the trial, Figure S1: Representative ^1^H NMR spectra of preterm infant urine before and after ADJ fortification, Figure S2: PC1 vs. PC2 scores plot of the PCA model built from ^1^H NMR spectra of urine samples collected from preterm infants before ADJ fortification.

## References

[1] Arslanoglu S., Boquien C.Y., King C., Lamireau D., Tonetto P., Barnett D., Bertino E., Gaya A., Gebauer C., Grovslien A. (2019). Fortification of human milk for preterm infants: Update and recommendations of the European Milk Bank Association (EMBA) Working Group on Human Milk Fortification. Front. Pediatr..

[2] Chan S.H.T., Johnson M.J., Leaf A.A., Vollmer B. (2016). Nutrition and neurodevelopmental outcomes in preterm infants: A systematic review. Acta Paediatr..

[3] Ong K.K., Kennedy K., Castañeda-Gutiérrez E., Forsyth S., Godfrey K.M., Koletzko B., Latulippe M.E., Ozanne S.E., Rueda R., Schoemaker M.H. (2015). Postnatal growth in preterm infants and later health outcomes: A systematic review. Acta Paediatr..

[4] Kumar R.K., Singhal A., Vaidya U., Banerjee S., Anwar F., Rao S. (2017). Optimizing nutrition in preterm low birth weight infants—Consensus summary. Front. Nutr..

[5] Kemp J.E., Wenhold F.A.M. (2016). Human milk fortification strategies for improved in-hospital growth of preterm infants. S. Afr. J. Clin. Nutr..

[6] Wagner J., Hanson C., Anderson-Berry A. (2014). Considerations in meeting protein needs of the human milk-fed preterm infant. Adv. Neonatal Care.

[7] Lenz E.M., Bright J., Knight R., Wilson I.D., Major H. (2004). A metabonomic investigation of the biochemical effects of mercuric chloride in the rat using 1H NMR and HPLC-TOF/MS: Time dependant changes in the urinary profile of endogenous metabolites as a result of nephrotoxicity. Analyst.

[8] Solanky K.S., Bailey N.J.C., Holmes E., Lindon J.C., Davis A.L., Mulder T.P.J., Van Duynhoven J.P.M., Nicholson J.K. (2003). NMR-based metabonomic studies on the biochemical effects of epicatechin in the rat. J. Agric. Food Chem..

[9] Stella C., Beckwith-Hall B., Cloarec O., Holmes E., Lindon J.C., Powell J., Van Der Ouderaa F., Bingham S., Cross A.J., Nicholson J.K. (2006). Susceptibility of human metabolic phenotypes to dietary modulation. J. Proteome Res..

[10] Walsh M.C., Brennan L., Pujos-Guillot E., Sébédio J.L., Scalbert A., Fagan A., Higgins D.G., Gibney M.J. (2007). Influence of acute phytochemical intake on human urinary metabolomic profiles. Am. J. Clin. Nutr..

[11] Johnson M.J., Wootton S.A., Leaf A.A., Jackson A.A. (2012). Preterm birth and body composition at term equivalent age: A systematic review and meta-analysis. Pediatrics.

[12] Ulaszewska M.M., Weinert C.H., Trimigno A., Portmann R., Andres Lacueva C., Badertscher R., Brennan L., Brunius C., Bub A., Capozzi F. (2019). Nutrimetabolomics: An integrative action for metabolomic analyses in human nutritional studies. Mol. Nutr. Food Res..

[13] Moltu S.J., Sachse D., Blakstad E.W., Strømmen K., Nakstad B., Almaas A.N., Westerberg A.C., Rønnestad A., Brække K., Veierød M.B. (2014). Urinary metabolite profiles in premature infants show early postnatal metabolic adaptation and maturation. Nutrients.

[14] Cesare Marincola F., Corbu S., Lussu M., Noto A., Dessì A., Longo S., Civardi E., Garofoli F., Grenci B., Mongini E. (2016). Impact of early postnatal nutrition on the NMR urinary metabolic profile of infant. J. Proteome Res..

[15] Alexandre-Gouabau M.C., Moyon T., David-Sochard A., Fenaille F., Cholet S., Royer A.L., Guitton Y., Billard H., Darmaun D., Rozé J.C. (2019). Comprehensive preterm breast milk metabotype associated with optimal infant early growth pattern. Nutrients.

[16] Morniroli D., Dessì A., Giannì M.L., Roggero P., Noto A., Atzori L., Lussu M., Fanos V., Mosca F. (2019). Is the body composition development in premature infants associated with a distinctive nuclear magnetic resonance metabolomic profiling of urine?. J. Matern. Neonatal Med..

[17] Shoji H., Shimizu T. (2019). Effect of human breast milk on biological metabolism in infants. Pediatr. Int..

[18] Bertino E., Cavallarin L., Cresi F., Tonetto P., Peila C., Ansaldi G., Raia M., Varalda A., Giribaldi M., Conti A. (2019). A novel donkey milk-derived human milk fortifier in feeding preterm infants: A randomized controlled trial. J. Pediatr. Gastroenterol. Nutr..

[19] Bertino E., Gastaldi D., Monti G., Baro C., Fortunato D., Garoffo L.P., Coscia A., Fabris C., Mussap M., Conti A. (2010). Detailed proteomic analysis on DM: Insight into its hypoallergenicity. Front. Biosci..

[20] Coscia A., Bertino E., Tonetto P., Peila C., Cresi F., Arslanoglu S., Moro G.E., Spada E., Milani S., Giribaldi M. (2018). Nutritional adequacy of a novel human milk fortifier from donkey milk in feeding preterm infants: Study protocol of a randomized controlled clinical trial. Nutr. J..

[21] Scalabre A., Jobard E., Demède D., Gaillard S., Pontoizeau C., Mouriquand P., Elena-Herrmann B., Mure P.Y. (2017). Evolution of newborns’ urinary metabolomic profiles according to age and growth. J. Proteome Res..

[22] Diaz S.O., Pinto J., Barros A.S., Morais E., Duarte D., Negrao F., Pita C., Almeida M.D.C., Carreira I.M., Spraul M. (2016). Newborn urinary metabolic signatures of prematurity and other disorders: A case control study. J. Proteome Res..

[23] Eriksson L., Trygg J., Wold S. (2008). CV-ANOVA for significance testing of PLS and OPLS^®^ models. A J. Chemom. Soc..

[24] Bertino E., Giribaldi M., Cester E.A., Coscia A., Trapani B.M., Peila C., Arslanoglu S., Moro G.E., Cavallarin L. (2017). New human milk fortifiers for the preterm infant. J. Pediatr. Neonatal Individ. Med..

[25] Marincola F.C., Dessì A., Pattumelli M.G., Corbu S., Ossicini C., Ciccarelli S., Agostino R., Mussap M., Fanos V. (2015). 1H NMR-based urine metabolic profile of IUGR, LGA, and AGA newborns in the first week of life. Clin. Chim. Acta.

[26] Gubhaju L., Sutherland M.R., Black M.J. (2011). Preterm birth and the kidney: Implications for long-term renal health. Reprod. Sci..

[27] Lionetti L., Cavaliere G., Bergamo P., Trinchese G., De Filippo C., Gifuni G., Gaita M., Pignalosa A., Donizzetti I., Putti R. (2012). Diet supplementation with donkey milk upregulates liver mitochondrial uncoupling, reduces energy efficiency and improves antioxidant and antiinflammatory defences in rats. Mol. Nutr. Food Res..

[28] Borum P.R. (1995). Carnitine in neonatal nutrition. J. Child Neurol..

[29] Penn D., Dolderer M., Schmidt-Sommerfeld E. (1987). Carnitine concentrations in the milk of different species and infant formulas. Neonatology.

[30] Melegh B. (1990). Carnitine supplementation in the premature. Neonatology.

[31] Van Aerde T. (2004). In preterm infants, does the supplementation of carnitine to parenteral nutrition improve the following clinical outcomes: Growth, lipid metabolism and apneic spells? Part A: Evidence-based answer and summary. Paediatr. Child Health.

[32] Van Aerde J.E. (2004). In preterm infants, does the supplementation of carnitine to parenteral nutrition improve the following clinical outcomes: Growth, lipid metabolism and apneic spells? Part B: Clinical commentary. Paediatr. Child Health.

[33] Sundekilde U.K., Downey E., O’Mahony J.A., O’Shea C.A., Ryan C.A., Kelly A.L., Bertram H.C. (2016). The effect of gestational and lactational age on the human milk metabolome. Nutrients.

[34] Boehm G., Borte M., Bellstedt K., Moro G., Minoli I. (1993). Protein quality of human milk fortifier in low birth weight infants: Effects on growth and plasma amino acid profiles. Eur. J. Pediatr..

[35] Dos Santos S.C., de Figueiredo C.M., de Andrade S.M.O., Palhares D.B. (2007). Plasma amino acids in preterm infants fed different human milk diets from a human milk bank. E-SPEN Eur. E-J. Clin. Nutr. Metab..

[36] Szajewska H. (2007). Extensive and partial protein hydrolysate preterm formulas. J. Pediatr. Gastroenterol. Nutr..

[37] Rigo J., Senterre J. (1994). Metabolic balance studies and plasma amino acid concentrations in preterm infants fed experimental protein hydrolysate preterm formulas. Acta Paediatr..

[38] Polberger S.K., Axelsson I.E., Räihä N.C. (1990). Urinary and serum urea as indicators of protein metabolism in very low birthweight infants fed varying human milk protein intakes. Acta Paediatr. Scand..

[39] Pozzo L., Cirrincione S., Russo R., Karamać M., Amarowicz R., Coscia A., Antoniazzi S., Cavallarin L., Giribaldi M. (2019). Comparison of oxidative status of human milk, human milk fortifiers and preterm infant formulas. Foods.

[40] He X., Parenti M., Grip T., Domellöf M., Lönnerdal B., Hernell O., Timby N., Slupsky C.M. (2019). Metabolic phenotype of breast-fed infants, and infants fed standard formula or bovine MFGM supplemented formula: A randomized controlled trial. Sci. Rep..

[41] Cresi F., Maggiora E., Pirra A., Tonetto P., Rubino C., Cavallarin L., Giribaldi M., Moro G.E., Peila C., Coscia A. (2020). Effects on gastroesophageal reflux of donkey milk-derived human milk fortifier versus standard fortifier in preterm newborns: Additional data from the fortilat study. Nutrients.

