# Supplementary material for: Urinary Metabolomic Profile of Preterm Infants Receiving Human Milk with Either Bovine or Donkey Milk-Based Fortifiers
Source: Nutrients. 2020 Jul 27;12(8):2247. doi: 10.3390/nu12082247 (PMC7468788; doi:10.3390/nu12082247)
Supplement: Supplementary file 1 [file nutrients-12-02247-s001.pdf]

SUPPORTING INFORMATION

Urinary metabolomic profile of preterm infants receiving human milk with either bovine- or donkey milk-based fortifiers

Marzia Giribaldi [1], Chiara Peila [2], Alessandra Coscia [2]*, Laura Cavallarin [3], Sara Antoniazzi [3], Sara Corbu [4], Giulia Maiocco [2], Stefano Sottemano [2], Francesco Cresi [2], Guido E. Moro [5], Enrico Bertino [2], Vassilios Fanos [6], Flaminia Cesare Marincola [4].

1 CREA Research Centre for Engineering and Agro-Food Processing, 10135 Turin, Italy; marzia.giribaldi@crea.gov.it

2 Neonatal Unit, University of Turin, City of Health and Science of Turin, 10126 Turin, Italy; enrico.bertino@unito.it; alessandra.coscia@unito.it; francesco.cresi@unito.it, stefano.sottemano@unito.it, giulia.maiocco@edu.unito.it, chiara.peila@unito.it

3 Institute of the Science of Food Production - National Research Council, 10095 Grugliasco (TO), Italy; laura.cavallarin@ispa.cnr.it, sara.antoniazzi@ispa.cnr.it

4 Department of Chemical and Geological Sciences, Cittadella Universitaria, University of Cagliari, 09042 Monserrato, Cagliari, Italy. flaminia@unica.it

5 Neonatal Intensive Care Unit, Neonatal Pathology and Neonatal Section, Azienda University Polyclinic, University of Cagliari, 09124 Cagliari, Italy. sari.crb@gmail.com; vafanos@tin.it

6 Italian Association of Human Milk Banks (AIBLUD), 20126 Milan, Italy; guidoemoro@tiscali.it

* Correspondence: alessandra.coscia@unito.it;

M.G and C.P. equally contributed to this paper.

Table S1. Macronutrient composition of the multi-component fortifiers and the protein concentrates derived from either bovine milk (B-HMF) or donkey milk (D-HMF). Values per 100 g of product.

	**B-HMF**	**D-HMF**
Multicomponent fortifier		
Protein g (Nx6.25)	20.0	22.5
Carbohydrate g	66.0	59.0
of which:		
Lactose g	6.0	59.0
Maltodextrin g	60.0	0.0
Fat g	0.4	3.6
Energy:		
Kcal	385	390
kcal/g protein	18.8	15.6
Calcium mg	1500	938
Phosphate mg	900	734
Osmolality mOsm/Kg	453	441
Protein concentrate		
Protein g (Nx6.25)	88.5	43.0
Carbohydrate g	<1.5	33.5
Fat g	≤2.0	6.1
Energy:		
Kcal	370	418
kcal/g protein	4.2	9.7
Calcium mg	1350	1650
Phosphate mg	700	1150

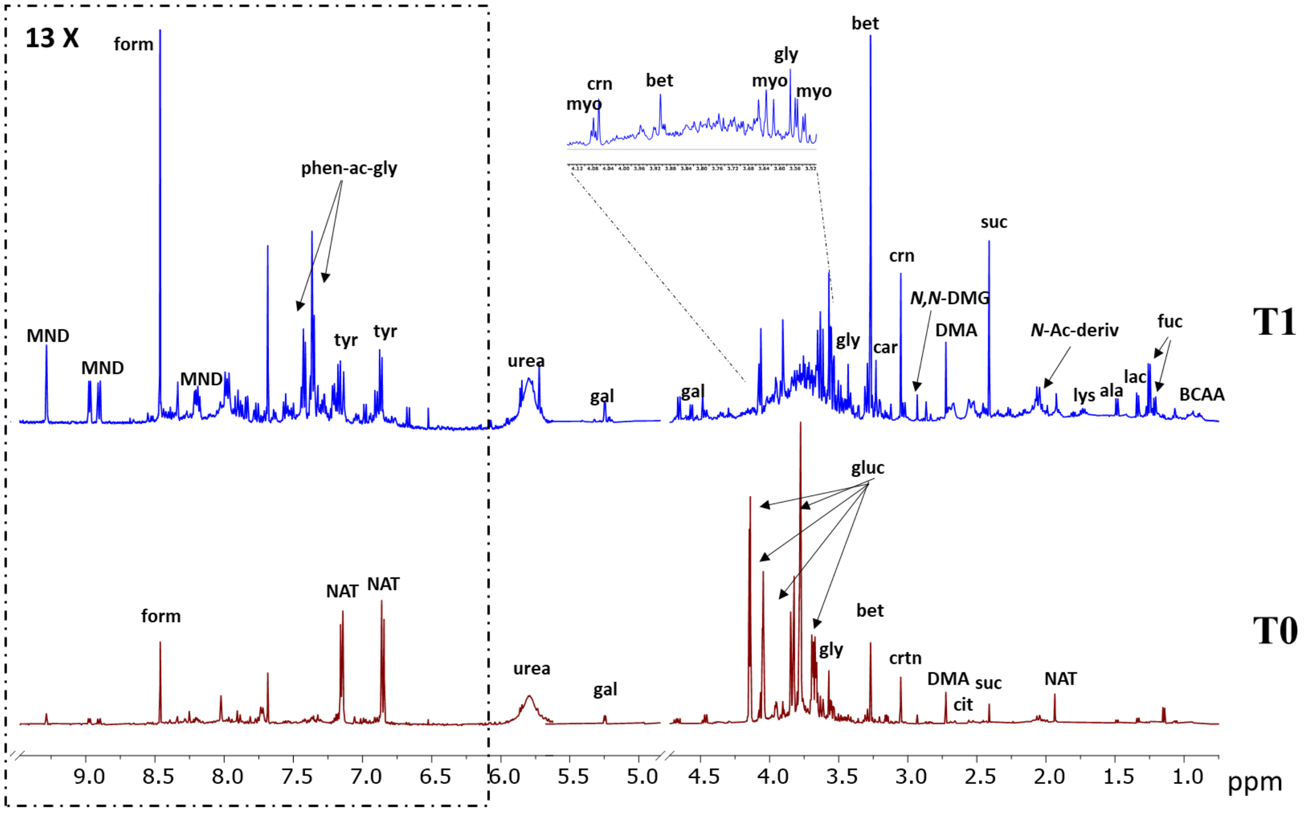

Figure S1. Representative ^{1}H NMR spectra of preterm infant urine collected before (T0) and after 21 days of ADJ fortification (T1). Abbreviations: ac, acetic acid; ala, alanine; BCAA, branched-chain amino acids; bet, betaine; car, carnitine; cit, citric acid; crn, creatinine; DMA, dimethylamine; form, formic acid; fuc, fucose; gal, galactose; gly, glycine; gluc, gluconate; lac, lactate; lys, lysine; myo, myo-inositol; MND, 1-methylnicotinamide, *N*-Ac-deriv, *N*-acetil derivatives; NAT, *N*-acetylthyrosine; *N*,*N*–DMG, *N*,*N*-dimethylglycine: phen-ac-gly, phenylacetyilglycine; suc, succinic acid; tyr, tyrosine.

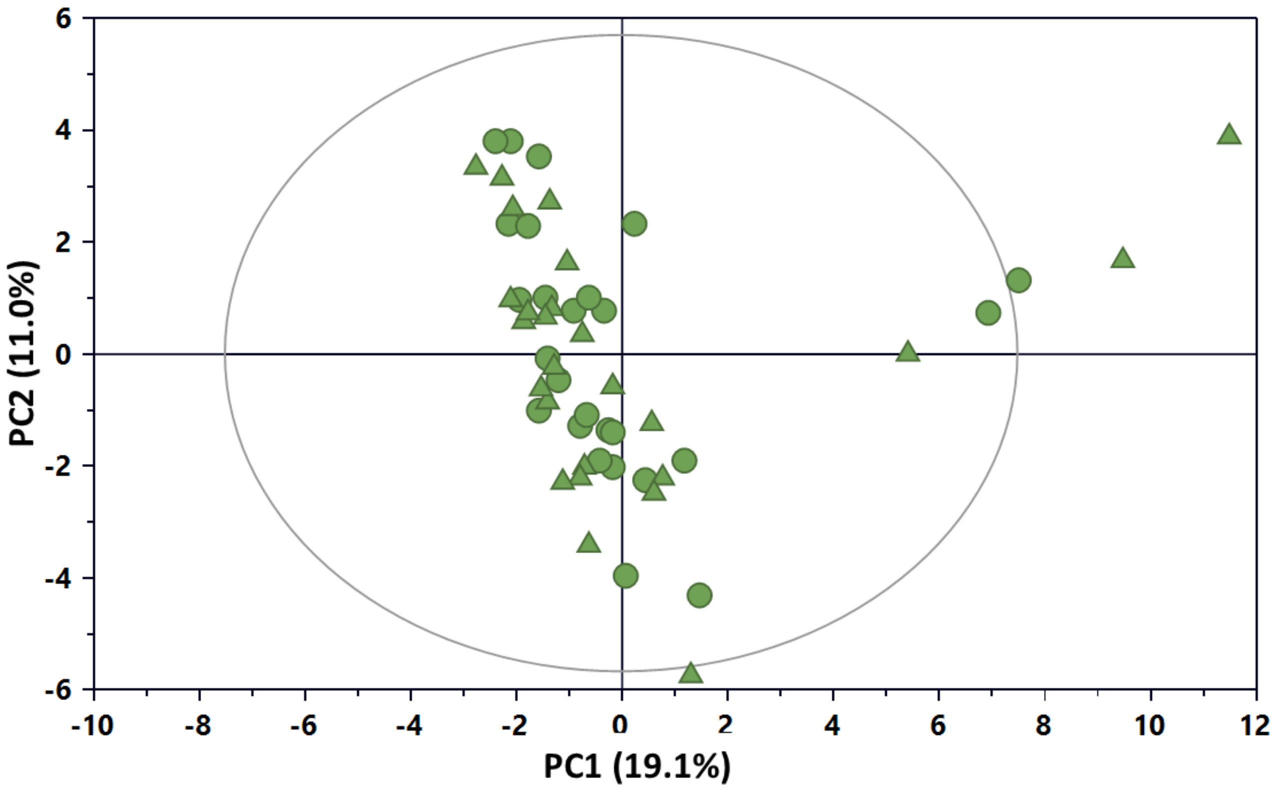

Figure S2. PC1 *vs* PC2 scores plot of the PCA model built with urine samples collected from preterm infants before ADJ fortification: o, B-HMF (infants receiving commercial multicomponent fortifier and protein concentrate derived from bovine milk); △, D-HMF (infants receiving experimental multicomponent fortifier and protein concentrates derived from donkey milk) (R^2X=0.310; Q^2=0.201).
